# A Case Report on Hepatic Extramedullary Hematopoiesis as the Manifestation of Progression to Secondary Myelofibrosis in a Patient with Essential Thrombocytopenia

**DOI:** 10.3390/hematolrep14040040

**Published:** 2022-09-21

**Authors:** Kaitlin I. McArthur, Philip N. Papayanis, Michael H. K. Nguyen, Yahya Daneshbod, Mojtaba Akhtari

**Affiliations:** 1 School of Medicine, Loma Linda University, Loma Linda, CA 92354, USA; 2 Department of Hematology Oncology, Department of Internal Medicine, Loma Linda University Medical Center, Loma Linda, CA 92354, USA; 3 Department of Pathology, Loma Linda University Medical Center, Loma Linda, CA 92354, USA

**Keywords:** myelofibrosis, essential thrombocytopenia, extramedullary hematopoiesis

## Abstract

Myeloproliferative neoplasms (MPN), which include primary myelofibrosis (PMF) and essential thrombocytopenia (ET), are characterized by the clonal proliferation of mature blood cells as a result of the overactivation of the JAK/STAT pathway. Extramedullary hematopoiesis (EMH), a common complication of PMF, occurs due to the dysregulation of the bone marrow microenvironment. We report an interesting case of a 73-year-old female with a working diagnosis of ET who was found to have EMH in the liver on biopsy after she had newly onset elevated liver enzymes and her ET had progressed to secondary myelofibrosis. We conclude that in patients with MPN who have rising liver enzymes, EMH in the liver should be part of the differential diagnosis. In addition, we believe that EMH is a sign of progression from MPN to secondary myelofibrosis and that it is imperative for performing bone marrow aspiration and biopsy in order to reassess hematopoiesis and to look for bone marrow fibrosis as well as evidence of progression.

## 1. Introduction

Myeloproliferative neoplasms (MPN) include essential thrombocythemia (ET), polycythemia vera (PV), and primary fibrosis (PMF), which are BCR-ABL1-negative. They are characterized by the clonal proliferation of blood cells as a result of the over activation of the JAK/STAT pathway. A relatively common complication of PMF is extramedullary hematopoiesis (EMH), which typically presents as organomegaly [1].

In healthy adults, hematopoiesis is a process that occurs in the bone marrow. When hematopoiesis takes place in adults in any organ other than bone marrow, it is called EMH and can occur in conditions that disrupt the environment of the bone marrow and decrease its function, such as MPN and thalassemias. Dysregulation of the bone marrow microenvironment that leads to the abnormal trafficking of hematopoietic stem cells and progenitor cells that are then transferred to the spleen is one current theory about EMH in the spleens of patients with MPN [2]. EMH can occur in almost all parts of the body, but it most commonly presents in paravertebral thoracic regions or in the liver, spleen, and lymph nodes. These are locations where fetal erythropoiesis takes place, but this stops at birth [3]. In patients where this occurs, an elevated ALP has been suggested as a sign of liver EMH, which can be confirmed with a liver biopsy that will typically show hepatic myeloid metaplasia [4]. 

We report an interesting case of a 73-year-old female with a working diagnosis of ET who was found to have EMH in the liver on biopsy after she had newly onset elevated liver enzymes and her ET had progressed to secondary myelofibrosis. EMH should be considered a sign of MPN progression and indicates the need for a change in therapeutic modality. 

## 2. Case Presentation

A 73-year-old lady presented to clinic with progressive weakness, night sweats, and fatigue. She had a past medical history of stage IIA left breast cancer ER100%/PR-/HER2- oncotype score 16 for which she had received a lumpectomy six years prior and adjuvant radiation therapy with five years of anastrozole. She had also been diagnosed with ET six years prior and was subsequently managed with hydroxyurea 500 mg twice a day, which had been increased to 1500 mg daily at the time of presentation. In addition, low-dose aspirin 81 mg was administered. Other notable symptoms she presented with included decreased appetite, leg swelling, heat intolerance, and numbness in the fingers and toes. Six months before presentation, the patient was found to have a right renal vein thrombosis due to her underlying condition. At that time, CT showed an unremarkable spleen and multiple hypodense lesions in her liver. Four months later, her spleen was also found to be enlarged on CT. 

Subsequently a bone marrow biopsy and aspiration were performed, which demonstrated grade 2–3 fibrosis with mildly increased megakaryocytes and diffuse reticulum fibers (Figure S1). Trichrome staining found grade 3 markedly and diffusely increased collagen deposits (Figure S2). The JAK2 V617F mutation was detected as being 31% of the total JAK2 DNA. Cytogenic karyotype testing could not be conducted due to the samples failing to produce mitotic or interphase cells. The patient was then hospitalized to work up hyperbilirubinemia and underwent a liver core biopsy that found EMH in the liver, predominantly within the sinusoidal spaces. The patient was also found to have elevated alkaline phosphatase (Table S3). Portal tracts did not demonstrate significant inflammation, and the bile ducts and vessels appeared normal. No significant fibrosis, alpha1 anti-tryptase deficiency, or abnormal iron storage were found. She was diagnosed with post-essential thrombocythemia myelofibrosis.

## 3. Discussion

In ET, the frequency of progression to secondary myelofibrosis is roughly 0.8–4.9% at 10 years and 4–11% at 15 years [5]. The presence of secondary myelofibrosis is a significant finding indicating the progression of this malignant disease. Our patient had the JAK 2 V617F mutation, which is associated with splenic hematopoiesis in MPN. The mutated JAK2 V617F protein is thought to be a constitutively activated cytoplasmic tyrosine kinase that mediates myeloproliferation via the JAK2/STAT5 signaling pathways. In addition, in patients who are positive for JAK2 V617F, there is an overexpression of Bcl-xL, an antiapoptotic protein, which may be implicated in EMH and is thought to play an important role in cell cytokine signaling, and its mutation can lead to an increased cytokine response [6]. Our patient presented with worsening splenomegaly, which was a sign of progression to secondary myelofibrosis. The presentation of EMH should prompt physicians to reassess MPN patients for progression to myelofibrosis and to reevaluate the appropriate therapeutic intervention since there are newly approved medications for MPN [7,8]. 

In the case of our patient, she had ET that had progressed to MF. Bone marrow aspiration and biopsy are necessary for the diagnosis of progression, as secondary MF is classically distinguished from ET by the presence of reticulin or collagen fibers and megakaryocyte hyperplasia in the bone marrow [9]. This has therapeutic implications, as, for example, ruxolitinib, fedratinib, and parctitinibare have been approved for use in a first-line setting for intermediate and high-risk PMF and post ET and PV myelofibrosis but not for ET [10,11]. 

In patients with PMF, there is evidence showing that elevated liver enzymes, most specifically alkaline phosphatase (ALP), can be used as a prognostic indicator for mortality in PMF. This elevation is thought to be due to EMH in the liver [11,12]. Given very similar pathologies, we can hypothesize that the same is likely true for other MPN, such as ET and PV. In this case, the patient presented with EMH in the liver and had elevated total bilirubin, ALT, AST, and ALP without any other underlying liver disease to explain these abnormalities. Of note, one study preformed liver biopsies in 22 patients with PMF and found hepatic myeloid metaplasia in all of them, with elevated ALP being the most frequent enzyme abnormality in these patients. This suggests that, as in PMF, the elevation of liver enzymes in a patient with MPN warrants further investigation for potential progression to secondary myelofibrosis with EMH, though further research is needed to determine clinical implications [4].

## 4. Conclusions

We conclude that in patients with MPN who have rising liver enzymes, EMH in the liver should be part of the differential diagnosis. In addition, we believe that EMH is a sign of progression to secondary myelofibrosis and that it is imperative for performing bone marrow biopsy and aspiration and for the evaluation of hematopoiesis and bone marrow fibrosis. 

## Data Availability

Not applicable.

## Supplementary Materials

The following supporting information can be downloaded at https://www.mdpi.com/article/10.3390/hematolrep14040040/s1: Figure S1: Bone marrow biopsy; Figure S2: Trichrome bone marrow biopsy; Table S3: Patient’s enzyme results.
