# Supplementary material for: A Case Report on Hepatic Extramedullary Hematopoiesis as the Manifestation of Progression to Secondary Myelofibrosis in a Patient with Essential Thrombocytopenia
Source: Hematol Rep. 2022 Sep 21;14(4):286–9. doi: 10.3390/hematolrep14040040 (PMC9590059; doi:10.3390/hematolrep14040040)
Supplement: Supplementary file 1 [file hematolrep-14-00040-s001.zip › hematolrep-1728080-supplementary.pdf]

Supplementary Materials:

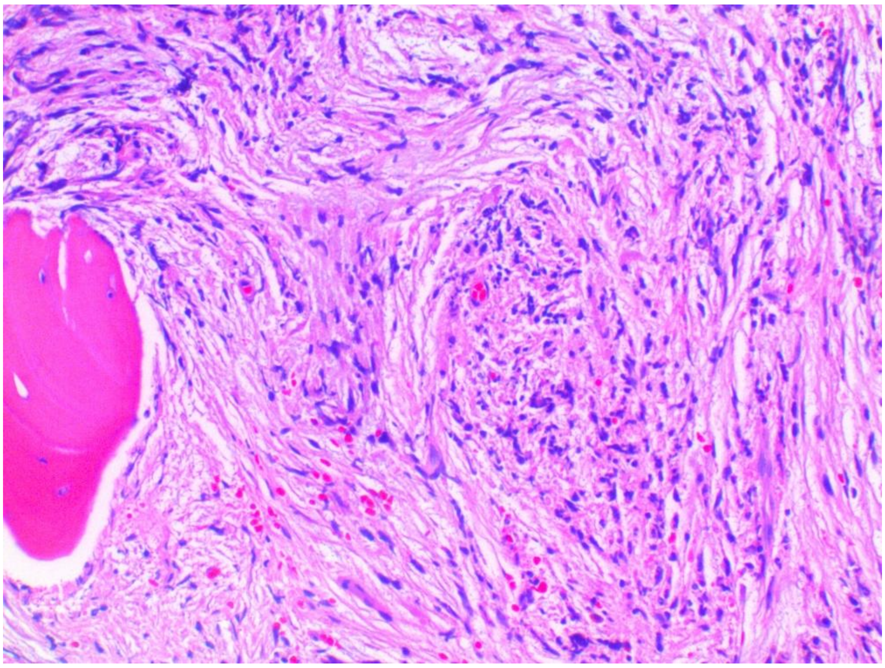

Figure S1. H&E bone marrow biopsy. Bone marrow shows diffuse fibrosis replacing the marrow's hematopoietic elements and mildly increased megakaryocytes (hematoxylin and eosin, x400).

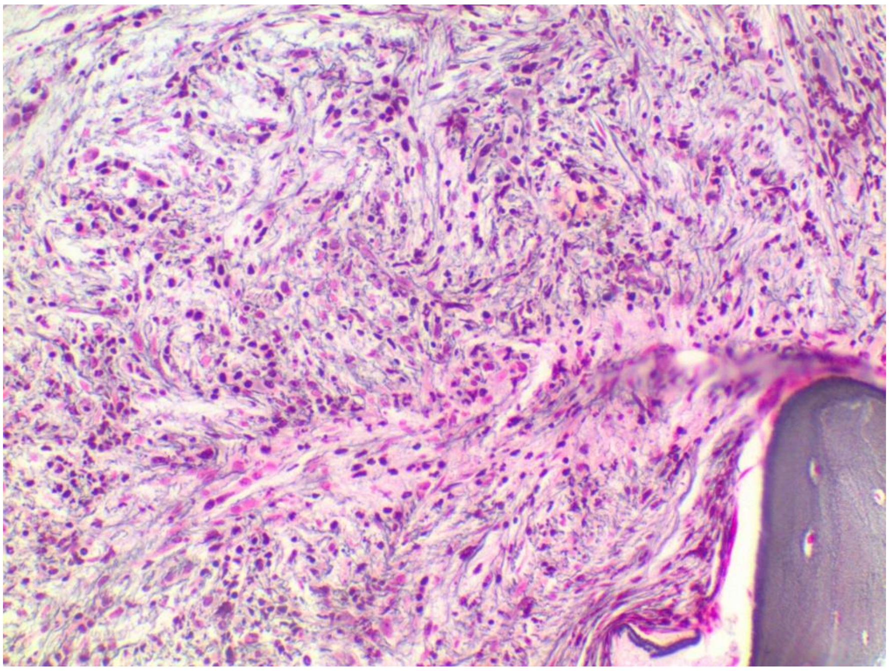

Figure S2. Reticulin bone marrow biopsy. Reticulin staining shows a diffuse and dense increase in the reticulin fibers and coarse collagen fibers (reticulin stain, x400).

Table S3. Patient's Enzyme Results: Grade 3 markedly and diffusely increased collagen deposits. Trichome stain, 20x.

Date	Alkaline Phosphatase	AST	ALT	Total Bilirubin
8/30/2019	54	24	24	1.2
10/14/2019	59	23	23	0.9
6/8/2020	58	20	19	0.8
10/29/2020	89	31	21	1.2
11/5/2020	93	28	21	0.9
11/13/2020	100	33	32	1.1
12/14/2020	81	25	19	1
1/20/2021	64	23	13	1.1
3/10/2021	71	21	12	1.4
4/7/2021	80	26	15	1.8
5/14/2021	116	27	13	2
6/10/2021	178	34	23	1.7
7/28/2021	361	62	51	2.8
8/3/2021	487	181	95	3.5
8/5/2021	524	48	49	3.1
8/6/2021	357	39	37	3
8/11/2021	395	57	39	2.8
8/19/2021	402	69	44	2.2
8/24/2021	330	51	42	2
9/2/2021	298	110	87	1.5
9/8/2021	363	89	105	1.3
9/23/2021	309	75	71	1.7
10/7/2021	283	50	58	1.8
10/16/2021	463	76	81	2.1
10/21/2021	497	70	74	1.5
10/28/2021	343	38	32	1.3
11/1/2021	226	49	35	1.4
11/3/2021	245	53	37	1.6
11/10/2021		54	41	1.4
